# Evaluation of In Vitro Inhibition of *β*-Hematin Formation: A Step Towards a Comprehensive Understanding of the Mechanism of Action of New Arylamino Alcohols

**DOI:** 10.3390/microorganisms12122524

**Published:** 2024-12-07

**Authors:** Céline Damiani, Floriane Soler, Yohann Le Govic, Anne Totet, Guillaume Bentzinger, Anne Bouchut, Romain Mustière, Patrice Agnamey, Alexandra Dassonville-Klimpt, Pascal Sonnet

**Affiliations:** 1 Agents Infectieux, Résistance et Chimiothérapie (AGIR), UR 4294, Université de Picardie Jules Verne, 1 rue des Louvels, 80037 Amiens, France; legovic.yohann@chu-amiens.fr (Y.L.G.); totet.anne@chu-amiens.fr (A.T.); guillaume.bentzinger@u-picardie.fr (G.B.); anne.bouchut@u-picardie.fr (A.B.); romain.mustiere@u-picardie.fr (R.M.); p.agnamey@u-picardie.fr (P.A.); alexandra.dassonville@u-picardie.fr (A.D.-K.); pascal.sonnet@u-picardie.fr (P.S.); 2 Laboratoire de Parasitologie et Mycologie, Centre de Biologie Humaine, CHU Amiens-Picardie, 1 Rond-Point du Pr Cabrol, 80054 Amiens, France; soler.floriane@gmail.com

**Keywords:** *Plasmodium falciparum*, hemozoin, *β*-hematin, cytotoxicity, arylamino alcohols, mechanisms of action

## Abstract

Currently, artemisinin-based combination therapy is recommended as first-line treatment of uncomplicated *falciparum* malaria. Arylamino alcohols (AAAs) such as mefloquine (MQ) are the preferred partner drugs due to their longer half-life, reliable absorption and strong antimalarial activity. However, the mode of action of MQ remains poorly understood and its neurotoxicity limits its use. Furthermore, the emergence of drug-resistant parasites requires development of new antimalarial drugs. The aim of this study was to evaluate the *β*-hematin inhibition capacity of three pairs of enantiopure AAAs **1–3** (***a*/*S*** and ***b*/*R***) derived from MQ or enpiroline (ENP), a pyridine-based MQ analog with strong antimalarial activity. Inhibition of *β*-hematin—the synthetic counterpart of hemozoin formation—was determined for each compound. Antimalarial activity against W2 and 3D7 *Plasmodium falciparum* strains as well as percentages of inhibition of *β*-hematin formation were compared to those of reference molecules, i.e., chloroquine (CQ), MQ and ENP. Furthermore, a cytotoxicity study on the human-derived hepatocarcinoma cell line HepG2 was performed. With high antimalarial activity, stronger ability to inhibit *β*-hematin formation and low cytotoxicity, AAAs **1a-b** and **2a** are the most promising. These findings provide a better understanding of their potential mechanisms of action and may pave the way toward developing new lead compounds.

## 1. Introduction

**Figure 1:**
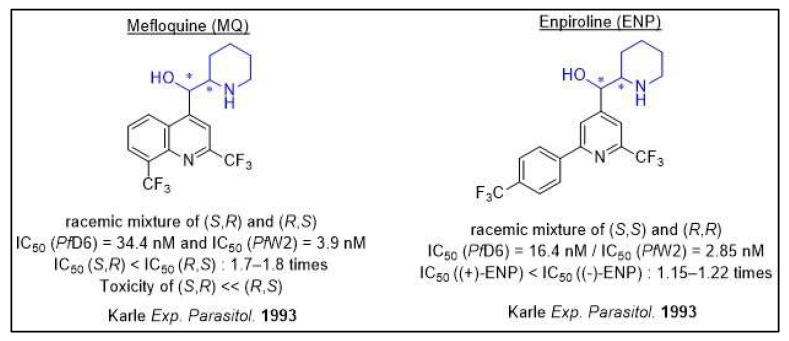
Structures of mefloquine (MQ) and enpiroline (ENP) enantiomers [13]. * stereogenic center.

**Scheme 1:**
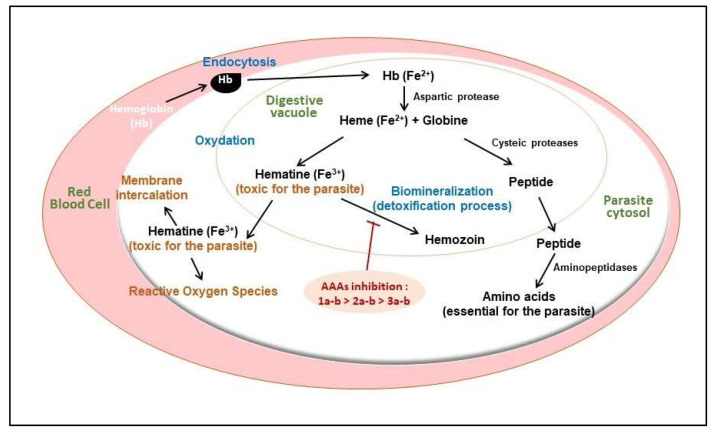
Schematic representation of the hemoglobin degradation process in *Plasmodium falciparum* and of the potential target of new AAAs.

Malaria remains one of the most significant infectious diseases in the world. In 2022, there were a total of 249 million new cases of malaria worldwide and 608,000 deaths, 76% of which were children under five [1]. *Plasmodium falciparum* (*Pf*) is the causative agent of the most dangerous form of the disease in humans. Nutrients and ions essential for the parasite’s growth and development are located in the host’s red blood cells (RBC). During intraerythrocytic growth, trophozoite stages of *Pf* can digest up to 80% of the RBC’s hemoglobin content to source amino acids required for protein production [2]. Hemoglobin is then degraded by proteases within the parasite’s acidic digestive vacuole (DV) into two entities, namely heme and globin. Globin is cleaved by enzymes into amino acids, essential for parasite metabolic functions and growth. Heme is initially oxidized into hematin, a toxic ferriprotoporphyrin IX (Fe(III)PPIX) whose accumulation can lead to *Plasmodium* death through reactive oxygen species (ROS) production. To avoid toxicity, *Plasmodium* parasites neutralize hematin into inert and insoluble hemozoin (Hz) crystals. Hz, whose synthetic counterpart is called *β*-hematin, represents an attractive target for common antimalarial drugs since aminoquinolines like chloroquine (CQ) and arylamino alcohols (AAAs) such as mefloquine (MQ, Figure 1) interfere with the crystallization process (Scheme 1) [3,4]. According to different studies [5,6,7], CQ and MQ are able to inhibit in vitro hemozoin formation by up to 90% and from 6% to 70%, respectively. The inhibition of hemozoin formation is the main mechanism of action of CQ. MQ has several targets within the parasites (including disruption of protein synthesis, impairment of lipid-binding proteins function and the induction of apoptosis) and is also able to prevent hemozoin formation even though it is not its main mechanism of action [8,9,10,11,12]. The mechanism of action of enpiroline (ENP, Figure 1) is not understood; notably, there is no data on the percentage of inhibition of *β*-hematin formation.

Nowadays, due to the parasite’s resistance to antimalarial drugs, and especially against artemisinin and its derivatives, agents displaying innovative chemistry have to be developed. Owing to their schizonticidal activity and good pharmacokinetic properties such as a long half-life, AAAs are interesting partners for artemisinin-based combination therapies (ACTs) [14]. Mainly used in combination with artesunate, MQ is one of the WHO Model List of Essential Medicines and has been commercially available since the 1970s as a racemic mixture of erythro enantiomers. However, MQ exhibits neurotoxic effects by disrupting calcium ion (Ca^2+^) homeostasis, resulting in a massive release of Ca^2+^ and ROS production [15,16]. Furthermore, it has been shown that (−)-*RS*-MQ enantiomer accumulates strongly in the central nervous system [17,18]. High affinity for brain adenosine receptors suggests its implication in neurological events associated with MQ usage [19]. In contrast, better antimalarial efficiency and lower neurotoxicity have been reported for (+)-*SR*-MQ enantiomer. To avoid adverse effects as well as to increase MQ antiplasmodial activity, the synthesis of quinoline analogs with an open-chain has been proposed [20].

**Figure 2:**
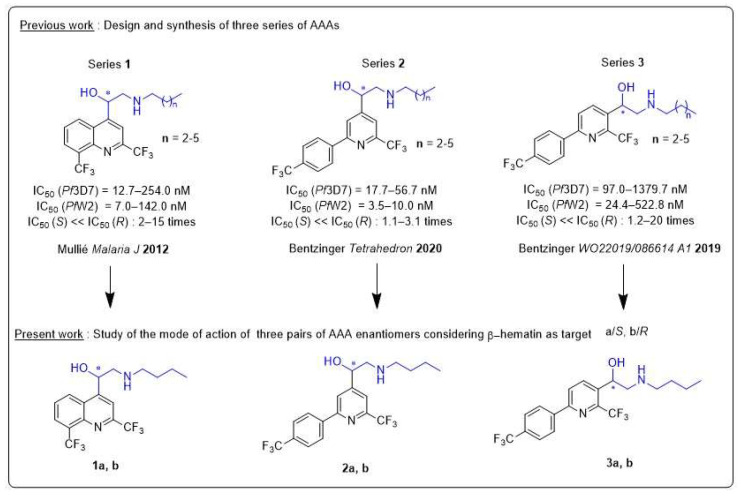
Structure of the three Series **1**, **2** and **3** previously synthesized and structure of (*S*)-enantiomers **1a, 2a** and **3a** and their (*R*)-counterparts **1b**, **2b** and **3b** selected for this study [6,22]. * stereogenic center.

In this context, we developed enantiopure analogs with an open-chain [6,21,22]. Thus, novel enantiopure MQ analogs (Series **1**, Figure 2) with a linear alkyl chain (C4-C7, n = 2–5) have been synthesized in five steps with good yields and excellent enantiomeric excesses. Their antimalarial activities ranged from 12.7 to 254.0 nM against the MQ-reduced susceptibility strain *Pf*3D7, and from 7.0 to 142.0 nM against the CQ-resistant strain *Pf*W2. In addition, (*S*)-enantiomers were 2–15 times more active than their (*R*)-counterpart, depending on the side chain length [6]. Then, focusing on the role of the aromatic ring, we dissociated the quinoline core to 6-phenylpyridines of Series **2** (Figure 2), considered as ENP analogs. The newly synthesized pyridines were more active than their quinoline counterparts (IC_50 (*Pf*3D7)_ = 17.7–56.7 nM and IC_50 (*Pf*W2)_ = 3.5–10.0 nM), but clearly showed fewer differences between (*S*)- and (*R*)- enantiomers (1.1–3.1 times). From these 4-aminoalcohol pyridine derivatives, the alkyl chain position was modified to afford the corresponding 3-aminoalcohol pyridines (Series **3**, Figure 2). The resulting compounds showed weaker antimalarial activity than compounds of Series **1** and **2**, with IC_50_ values ranging from 97.0 to 1379.7 nM against *Pf*3D7 and from 24.4 to 522.8 nM against *Pf*W2. However, eudysmic ratios were surprisingly higher than those of the other two Series (1.2–20 times), constantly in favor of (*S*)-enantiomer.

These previous works confirmed the interest in developing new enantiopure AAAs as MQ analogs (marketed drug), and ENP analogs (non-marketed drug) to fight against resistant strains of *Pf*. However, efforts should be made to better understand their mode of action, which could explain the differences in antimalarial activity observed in these three Series of AAAs (***a/S*** and ***b/R***). The aim of the present study was to evaluate the influence of the heterocyclic ring on the inhibition of hemozoin formation, which is assumed to be one of the main mechanisms of action of these compounds (Scheme 1). For this purpose, AAAs **1a-b**, **2a-b** and **3a-b** were selected, because the structure of their alkyl amino alcohol chain (shown in blue in Figure 2) confers potent antimalarial activities. The corresponding results would enable us to establish new structure–activity relationships (SAR) considering *β*-hematin (synthetic Hz analog) as target. The *β*-hematin inhibition assay was performed to evaluate the corresponding inhibition percentages of compounds **1a-b**, **2a-b** and **3a-b** and the three reference molecules CQ, MQ and ENP. To complete these data, a cytotoxicity study on the human-derived hepatocarcinoma cell line HepG2 was also carried out on the most active compounds to consider their selectivity index (SI).

## 2. Materials and Methods

### 2.1. Arylamino Alcohols Derivatives ***1a-b***, ***2a-b*** and ***3a-b***

The enantioselective synthesis of AAA derivatives **1a-b**, **2a-b** and **3a-b** was carried out according to the procedures previously described [23].

IC_50_s, corresponding to in vitro antimalarial activity against *Pf*W2 and *Pf*3D7, was previously determined for compounds **2a-b**, **3a-b** and reference molecules using the SYBR Green I fluorescence-based method. As compounds **1a-b** were previously screened using the hypoxanthine-tritied method [6], they were here re-assayed with the SYBR Green I fluorescence-based method described elsewhere [24,25] for comparison with the other two Series (**2** and **3**) and reference molecules. When possible, eudysmic ratios were calculated. The SYBR green I fluorescence-based method is detailed below.

Compounds were dissolved in DMSO and then diluted in sterile water in order to obtain a range of concentrations from 40 nM to 40 mM for the first screening against culture-adapted *Plasmodium falciparum* reference strains 3D7 and W2. The former strain is susceptible to CQ but displays a decreased susceptibility to MQ; the latter is considered resistant to CQ. These two strains were obtained from the collection of the National Museum of Natural History (Paris, France). The parasites were cultivated in RPMI medium (Sigma-Aldrich, Saint Quentin Fallavier, France) supplemented with 0.5% Albumax I (Life Technologies Corporation, Paisley, UK), hypoxanthine (Sigma-Aldrich, Saint Quentin Fallavier, France) and gentamicin (Sigma-Aldrich, Saint Quentin Fallavier, France) with human erythrocytes and were incubated at 37 °C with 5% CO_2_ [26]. The *P. falciparum* drug susceptibility test was carried out in 96-well flat-bottomed sterile plates in a final volume of 250 μL. After a 48 h incubation period with the drugs, quantities of DNA in treated and control cultures of parasites in human erythrocytes were quantified using the SYBR Green I (Sigma-Aldrich, Saint Quentin Fallavier, France) fluorescence-based method [27,28]. Briefly, after incubation, the plates were frozen at −20 °C until use. The plates were then thawed for 2 h at room temperature, and 100 μL of each homogenized culture was transferred to a well of a 96-well flat-bottomed sterile black plate (Sigma-Aldrich, Saint Quentin Fallavier, France) that contained 100 μL of the SYBR Green I lysis buffer (2xSYBR Green, 20 mM Tris base pH 7.5, 5 mM EDTA, 0.008% *w/v* saponin, 0.08% *w/v* Triton X-100). Negative controls treated with solvent (typically DMSO or H_2_O) and positive controls (CQ and MQ) were added to each set of experiments. Plates were incubated for 1 h at room temperature and then read on a fluorescence plate reader (Tecan Trading AG, Männedorf, Switzerland) using excitation and emission wavelengths of 485 and 535 nm, respectively. The concentrations at which the screening drug or antimalarial can inhibit 50% of parasitic growth (IC_50_s) were calculated from a sigmoid inhibition model Emax with an estimate of IC_50_s by non-linear regression (IC Estimator version 1.2) and were reported as means calculated from three independent experiments [29].

### 2.2. Inhibition of β-Hematin Formation

Inhibition of *β*-hematin formation of **1a-b**, **2a-b**, **3a-b** and reference molecules was assessed using the assay previously described in [5,30]. Stock solutions (20 mM) of CQ-phosphate were prepared in water while MQ and AAA derivatives were solubilized in MeOH/DMSO (4/1, *v*/*v*). Inhibition experiments were carried out over a concentration range of 0–2 mM (final concentration of the drug in wells). Briefly, 100 µL of a fresh 6.5 mM solution of hemin (Sigma-Aldrich, SaintQuentin Fallavier, France) dissolved in 0.2 M NaOH was mixed with 100 µL of 3 M sodium acetate, 25 µL of 17.4 M acetic acid and 25 µL of the tested drug or controls (water and MeOH/DMSO). Magnetic stirring of the solution was used to avoid sedimentation. After 24 h incubation at 37 °C under shaking, the supernatant resulting from centrifugation for 15 min at 3300× *g* was discarded. The pellet was washed twice with 200 µL DMSO. After a final wash with water, the pellet was dissolved in 200 µL with 0.1 M NaOH. After a further 1:30 dilution, absorption at 405 nm was read using a microplate photometer (Multiskan^®^ EX, Thermo Scientific, Waltham, MA, USA). CQ, MQ, ENP (considered as positive controls), water and MeOH/DMSO (considered as negative controls) were included in each experiment. Results were expressed as percentages of inhibition of *β*-hematin formation at 2 mM in comparison to the negative control result, calculated by the following formula [5]:

% inhibition = 100 × (1 − (OD molecule)/(OD control)).

Results were given as mean ± standard deviation of values obtained from three independent experiments and, when achievable, IC_50_ values were determined graphically.

Comparisons of percentages of inhibition of *β*-hematin formation were made using the Mann–Whitney signed rank test using Graphpad Prism 5.0 software. Statistical significance was defined as a value of *p* ≤ 0.05.

### 2.3. In Vitro Toxicity

The cytotoxicity of the most active compounds and reference molecules was evaluated with the MTT (3-(4,5-dimethylthiazol-2-yl)-2,5-diphenyltetrazolium bromide) assay adapted from [31]. Briefly, in 96-well plates, 100 µL of a cell culture adjusted to 25,000 cells/mL was dispensed and the plates were incubated at 37 °C in a humidified 5% CO_2_ atmosphere for 24 h to allow the cells to adhere to the bottom of the wells. After microscopic verification of the adherent cells, the culture medium was removed and replaced with 100 μL of medium with the compounds at various concentrations dissolved in DMSO (final concentration less than 0.5% *v*/*v*), and the plates were incubated for 72 h at 37 °C. Controls were DMSO in medium (0.5% *v*/*v*) without compound as blank. Then, the supernatant from each well was removed prior to the addition of 100 µL of 3-(4,5-dimethyl-2-thiazolyl)-2,5-diphenyl-2*H*-tetrazolium bromide (MTT) solution (0.5 mg/mL in culture medium) per well. After 2 h of incubation, the supernatant from each well was removed and 100 µL of DMSO was added to dissolve the resulting blue formazan crystals accumulated at the bottom of the wells. The plates were incubated for 5 min at 37 °C in a humidified 5% CO_2_ atmosphere. Then, the wells were homogenized by shaking the plates for 10 min with a microplate shaker. The absorbance was read using the Tecan’s Sunrise™ absorbance microplate reader at 570 nm with 630 nm as the reference wavelength. The 50% cytotoxic concentration (CC_50_) values were determined by nonlinear regression analysis processed on dose–response curves, using the Quest Graph™ IC_50_ Calculator1. Results were given as mean ± standard deviation of values obtained from three independent experiments. Selectivity index (SI) was calculated and defined as the ratio between CC_50_ values on HepG2 and IC_50_.

## 3. Results

Results of in vitro antimalarial activity against *Pf*W2 and *Pf*3D7 strains, inhibition of *β*-hematin formation and in vitro cytotoxicity results against HepG2 for **1–3** and reference molecules (CQ, MQ, ENP) are reported in Table 1 and Table 2.

ENP exhibited better antimalarial activity, whatever the *Pf* strain, compared with CQ and MQ (IC_50 (*Pf*W2)_ = 11.5 nM vs. 198.8 nM and 31.8 nM, respectively; IC_50 (*Pf*3D7)_ = 21.6 nM vs. 75.9 nM and 79.7 nM, respectively). In addition, **1a-b** were the more active compounds. In particular, they were more active than MQ on both *Pf*W2 and *Pf*3D7 (IC_50 (*Pf*3D7)_ = 15.1 nM and 15.1 nM vs. 79.7 nM, and IC_50 (*Pf*3W2)_ = 5 nM and 7.3 nM vs. 31.8 nM, respectively).

Considering the results of inhibition of *β*-hematin formation expressed in IC_50_ (mM), CQ was significantly more efficient in inhibiting *β*-hematin formation than all tested compounds except **1a-b**. By contrast, ENP and **3a-b** exhibited the highest IC_50_ > 2 mM. No statistically significant difference was observed between enantiomers **1a** and **1b** (1.17 vs. 1.31 mM, *p* > 0.05). However, **1a-b** displayed a significantly higher inhibition of *β*-hematin formation than its parental drug MQ (*p* = 0.0004 and 0.015, respectively).

Likewise, **2a-b** showed significantly higher inhibition of *β*-hematin formation compared to their parental drug ENP (*p* = 0.00016 and 0.013, respectively). Moreover, **2a-b** presented significantly higher inhibition than **3a-b** (IC_50_ = 1.45 and 1.77 vs. >2 mM, *p* < 0.05), but no significant difference was observed between enantiomers **2a** and **2b** (IC_50_ = 1.45 vs. 1.77 mM, *p* > 0.05) or between **3a** and **3b** (48.3% vs. 40.6% at 2 mM, *p* > 0.05).

Concerning in vitro cytotoxicity results against HepG2, **2a-b** were as cytotoxic as their respective parental drug ENP (CC_50_ at 4 and 4 vs. 4.4 µM, respectively), but their selectivity index (SI) was 87 and 70, respectively. The most active compounds, **1a-b**, were also among the less cytotoxic drugs and they were three to four times less cytotoxic than MQ (25.6 and 37.8 vs. 8.6 µM, respectively). Compound **1b** had the best SI, estimated at 2503.

## 4. Discussion

Regarding *β*-hematin as target, CQ is considered as the most potent molecule for inhibiting its formation. Indeed, CQ is known to inhibit in vivo hemozoin formation, defining its main action mode. In our study, its percentage of inhibition reached 86.9%, which is consistent with a previous report [4]. In contrast, the modes of action of MQ are still poorly understood, but it is believed to play a lesser role in inhibition of hemozoin formation [9,10,32,33,34]. This is consistent with our results, since IC_50_ of *β*-hematin formation for MQ was significantly higher than that of CQ (1.79 vs. 1.13 mM, *p* = 0.0004). Furthermore, the present study provides the first data concerning the percentage of inhibition of *β*-hematin formation for ENP (20.3%). Thus, ENP exhibited a significantly weaker *β*-hematin inhibition capacity but better antimalarial activity, whatever the *Pf* strain, compared with CQ and MQ. These results suggest that the slight structural difference that exists between MQ and ENP, i.e., the dissociation of the quinoline ring of MQ into 6-phenylpyridine in ENP, has a significant impact on the mode of action of this class of antimalarial drugs.

Compounds **1a-b** can be considered as open-MQ analogs as they possess a quinoline core with an open-butyl chain in place of the piperidine ring at the 4-position. However, these enantiomers are stronger for inhibited *β*-hematin formation than MQ, with IC_50_ at 1.17 and 1.31 mM, respectively, vs. 1.79 mM. In addition, compounds **1a-b** were more active than MQ on both *Pf*W2 and *Pf*3D7, with SI 15–23 times higher than that of their respective parental drug. Thus, the opening of the piperidine ring present in MQ could have an impact on both antimalarial activity and, to a lesser extent, the mode of action of these AAAs.

Compounds **2a-b** and **3a-b** have the same open butyl chain as **1a-b**, but the quinoline core of **1a-b** was dissociated into 6-phenylpyridine. Compounds **2a-b** and **3a-b** share the same heterocycle but differ from each other by the branched chain located either at the 4- or at the 3-position of the pyridine core. Inhibition of *β*-hematin formation for **2a-b** was significantly stronger than that of ENP. These results suggest that compound **2** may have a different mechanism of action than ENP. Interestingly, the antimalarial activity of **2a-b** was close to that of ENP against the *Pf*W2 strain (but not against *Pf*3D7) while **3a**-**b** were the least active compounds. Indeed, compared with ENP, the antimalarial activity of **3a-b** was 11 to 45 times weaker against *Pf*W2 and 22 to 63 times weaker against *Pf*3D7. Remarkably, the enantiomer with a (*S*)-absolute configuration **3a** was found to be significantly more active than its (*R*)-counterpart **3b**, as shown by eudysmic ratios of 4 and 2.9 against *Pf*W2 and *Pf*3D7, respectively. This difference in activity does not seem to be correlated with the inhibition of *β*-hematin because the percentages of inhibition of **3a** and **3b**, at 2 mM, were similar. These results suggest another target or a differential mode of transport for these enantiomers in the DV. Furthermore, antimalarial activity against both *Pf* strains and inhibition of *β*-hematin formation were stronger for compounds with an open-butyl chain located at the 4-position of the pyridine ring (**2a-b**) than for those branched at the 3-position (**3a-b**). Thus, SAR study on the open-butyl chain of compounds **2**–**3** indicates that the position of the alkyl chain has a greater impact on the antimalarial activity and the inhibition of *β*-hematin formation than the dissociation of the quinoline core. Altogether, these results show that the opening of the piperidine ring led to the formation of compounds **2a-b**, which were more active than and as toxic as ENP. AAAs **2a-b** seem to have different mechanisms of action from ENP and possess a stronger capacity to inhibit *β*-hematin formation. The AAAs **1**–**3**, along with the references MQ and ENP, are currently being evaluated across strains with different resistance profiles (*Pf*Dd2, *Pf*F32, *Pf*C580Y) to gain deeper insights into their mode of action and antimalarial spectrum.

## 5. Conclusions

Regarding hematin crystallization as an antiplasmodial target of interest, this work brought new SAR in the AAAs family. When comparing the piperidine compounds (MQ and ENP), the dissociation of the quinoline ring seems to be discriminant for in vitro *β*-hematin inhibition, suggesting a different mode of action for these two references. Contrastingly, regarding the 4-substituted open-butyl chain derivatives **1a-b** and **2a-b**, *β*-hematin formation is inhibited in the same way by quinoline and 6-phenylpyridine-based compounds. Moreover, when considering the 6-phenylpyridines **2a-b** and **3a-b**, the position of the alkyl chain appears important for both antimalarial activity and inhibition of *β*-hematin formation. Overall, in our study, the 4-substituted open-butyl chain compounds were the best inhibitors of *β*-hematin formation regardless of the heterocyclic core. Thus, **1a-b** and **2a** were the most active compounds on the two strains. In particular, they were more active than their parental drugs on the resistant strain *Pf*W2. Moreover, regarding their cytotoxicity profiles, **1a-b** are safer than MQ and **2a** is as toxic as ENP. In contrast to the piperidine ring opening, dissociation of the quinoline core is less discriminating for the inhibition of *β*-hematin formation. The data obtained in this study regarding the heme detoxification pathway represent a significant step towards a comprehensive understanding of the mechanism of action of the AAA derivatives. We are now considering further deciphering of this mechanism by assessing the transcriptomic and metabolomic responses of the parasite when cultured with these compounds. To fully characterize the mechanism of action, we also plan to identify mutations induced during ramping selection assays on the *Pf*W2 strain through a whole-genome sequencing approach.

## Figures and Tables

**Table 1:** In vitro antimalarial activity against Plasmodium falciparum W2 and 3D7 strains and eudysmic ratios for six arylamino alcohols (**1a-b**, **2a-b**, **3a-b**) and three reference molecules (chloroquine, mefloquine, enpiroline).

Compound	Config.		IC_50_ (nM) ^a,b^		Eudysmic Ratio ^c^	
			*Pf*W2 ^d^	*Pf*3D7 ^e^	*Pf*W2	*Pf*3D7
**Chloroquine**			198.8 ± 27.0	75.9 ± 3.0	NA	NA
**Mefloquine**			31.8 ± 1.0	79.7 ± 8.5	NA	NA
**Enpiroline**			11.5 ± 2.3	21.6 ± 1.4	NA	NA
**1**		**1a**	5.0 ± 0.3	15.1 ± 3.3	1.46 (*S*)	1
		**1b**	7.3 ± 0.9	15.1 ± 3.4		
**2**		**2a**	9.0 ± 0.4	45.8 ± 2.3	1.1 (*S*)	1.2 (*S*)
		**2b**	10.0 ± 0.7	56.7 ± 4.8		
**3**		**3a**	131.7 ± 10.3	479.0 ± 38.2	4.0 (*S*)	2.9 (*S*)
		**3b**	522.8 ± 95.8	1379.7 ± 992.8		

^a^ 50% inhibitory concentration (IC_50_) corresponds to the mean ± standard deviation from three independent experiments. ^b^ IC_50_s (nM) against *Pf*W2 and *Pf*3D7 of compounds **1**–**3** was determined using the SYBR Green I fluorescence-based method. ^c^ The eudysmic ratio was calculated using the following formula: IC_50_ eutomer/IC_50_ distomer. The eutomer configuration is between parentheses. ^d^ Resistant to CQ and sensitive to MQ. ^e^ Susceptible to CQ but displays a decreased susceptibility to MQ. NA: Not Applicable.

**Table 2:** Inhibition of *β*-hematin formation and in vitro cytotoxicity results against HepG2 for six arylamino alcohols (**1a-b**, **2a-b**, **3a-b**) and three reference molecules (chloroquine, mefloquine, enpiroline).

		Inhibition of *β*-Hematin Formation		Cytotoxicity Results Against HepG2	
Compound	Config.	Inhibition (%) ^a^ at 2 mM	IC_50_ (mM) ^a^	CC_50_ (µM) on HepG2 ^a^	Selectivity Index ^b^ HepG2/3D7
**Chloroquine**		86.9 ± 4.8	1.13 ± 0.20	24.1 ± 0.8	317
**Mefloquine**		61.3 ± 8.5	1.79 ± 0.09	8.6 ± 2.6	108
**Enpiroline**		20.3 ± ND ^c^	>2	4.4 ± 0.6	204
**1**	**1a**	81.8 ± 8.4	1.17 ± 0.32	25.6 ± 2.7	1695
	**1b**	71.3 ± 10.3	1.31 ± 0.54	37.8 ± 1.9	2503
**2**	**2a**	73.5 ± 18.0	1.45 ± 0.30	4.0 ± 0.9	87
	**2b**	54.0 ± 22.7	1.77 ± 0.22	4.0 ± 0.7	70
**3**	**3a**	48.3 ± ND ^c^	>2	11.6 ± 0.7	24
	**3b**	40.6 ± 28.3	>2	>100	>72

^a^ Mean ± standard deviation from three independent experiments. ^b^ Selectivity index was defined as the ratio between the CC_50_ value on the HepG2 cell line and the IC_50_ value against the *Pf*3D7 strain. ^c^ Results obtained in simplicate. ND: Not Determined.

## Data Availability

The original contributions presented in this study are included in the article. Further inquiries can be directed to the corresponding author.
